# Autophagy occurs in lymphocytes infiltrating Sjögren’s syndrome minor salivary glands and correlates with histological severity of salivary gland lesions

**DOI:** 10.1186/s13075-020-02317-6

**Published:** 2020-10-13

**Authors:** Serena Colafrancesco, Marta Vomero, Valentina Iannizzotto, Antonina Minniti, Cristiana Barbati, Francesca Arienzo, Linda Mastromanno, Tania Colasanti, Raffaella Izzo, Saba Nayar, Elena Pipi, Bruna Cerbelli, Carla Giordano, Francesco Ciccia, Fabrizio Conti, Guido Valesini, Francesca Barone, Roberta Priori, Cristiano Alessandri

**Affiliations:** 1 Dipartimento di Scienze Cliniche Internistiche, Anestesiologiche e Cardiovascolari, Sapienza University of Rome, Rome, Italy; 2 Rheumatology Research Group, Institute of Inflammation and Ageing, University of Birmingham, Birmingham, UK; 3 Dipartimento di Radiologia, Oncologia e Scienze Patologiche, Sapienza University of Rome, Rome, Italy; 4 Dipartimento di Medicina di Precisione, Rheumatology Unit, University of Campania “L. Vanvitelli”, Naples, Italy; 5 National Institute for Health Research (NIHR) Birmingham Biomedical Research Centre, University Hospitals Birmingham NHS Foundation Trust, UK and Sandwell and West Birmingham Trust, Birmingham, UK

**Keywords:** Autophagy, Lymphocytes, Sjögren’s syndrome, Minor salivary gland, LC3B, Atg5

## Abstract

**Backgrounds:**

The organization of minor salivary glands (MSG) infiltrates, in patients with Sjögren’s syndrome (SS), associates with disease severity and progression. Aberrant regulation of lymphocyte autophagy is involved in autoimmunity, and in previous work, we provided the first evidence of upregulated autophagy in CD4+ T cells infiltrating SS MSG. The aim of this study was to further explore autophagy in SS infiltrating and circulating lymphocytes and to investigate its role in disease histopathological progression.

**Methods:**

After collection of 20 SS MSG, the presence of lymphocyte aggregates (foci) and the formation of germinal center (GC)-like structures were observed by H&E and confirmed by immunohistochemistry. The expression of autophagy-related genes, Atg5 and MAP1LC3A, was detected by RT-PCR on microdissected salivary gland tissue and control tonsils. In MSG and tonsils, autophagic lymphocytes were identified by the detection of the autophagosome protein LC3B visualized as *LC3 puncta* staining by immunofluorescence. Peripheral blood autophagy was assessed by flow cytometry in SS and healthy controls (HC).

**Results:**

Real-time PCR demonstrated higher expression in the autophagy genes Atg5 and MAP1LC3A in MSG GCs as compared to both small foci (*p* = 0.0075, *p* = 0.0002) and GCs from tonsils (*p* = 0.0001, *p* = 0.0037). In MSG, *LC3 puncta* staining was detectable on both CD3+ and CD20+ lymphocytes; in tonsils, *LC3 puncta* was almost undetectable on all lymphocytes. Compared to HC (*n* = 20), flow cytometry did not reveal any increase of autophagy in SS circulating lymphocytes (*n* = 30).

**Conclusions:**

In SS MSG, lymphocytes’ autophagy is a feature of infiltrating T and B cells and is associated with histological severity. Interestingly, in MSG aberrant regulation of autophagy is detectable in GC-like structures possibly indicating its involvement in the development and persistence of the autoimmune process within the lesions.

## Key messages

Autophagy is a feature of infiltrating, but not circulating, T and B lymphocytes from patients with SS.

Activation of autophagy in lymphocytes infiltrating SS MSG mirrors disease histological severity.

Autophagy is upregulated in SS GC-like structures compared to the classic GCs inhabiting secondary lymphoid organs.

## Background

Autophagy is a cell-protective catabolic process aimed at eliminating damaged organelles, misfolded proteins, and intracellular pathogens [1]. Thanks to the ability to shape the adaptive immune response, deregulation of autophagy mechanisms seems implicated in the development of autoimmunity [2]. During stressful conditions, autophagy seems able to shape the adaptive immune response, orchestrating the regulation of lymphocyte survival, differentiation, and activation [3]. Several autophagy (Atg)-related proteins are implicated in this pathway, leading to the downstream conjugation of the microtubule-associated protein 1A/1B-light chain 3-I (LC3-I) with phosphatidylethanolamine and subsequent formation of LC3-II (LC3-phosphatidylethanolamine conjugate) [4]. LC3-II plays a crucial role in cargo selection and autophagosome biogenesis and is thereby widely utilized as a marker of autophagy activation [5]. LC3, which is identified by three different forms named LC3A, LC3B, and LC3C [6], is a structural protein of the autophagosomal membranes used as a biomarker of autophagy. Specifically, the cytoplasmic presence of LC3B, which is measured by immunofluorescence as an increase in punctate LC3 named “LC3 puncta” stain, is widely used to monitor autophagy [7, 8].

We previously investigated the engagement of autophagy in the pathogenesis of several autoimmune conditions, demonstrating a role in both promoting the exposure of immunogenic peptides and supporting immune cell survival in patients with rheumatoid arthritis [9]. Additionally, we demonstrated a natural resistance of T lymphocytes to autophagy in systemic lupus erythematous [10]. More recently, we provided the first evidence of autophagy mechanisms in CD4+ T lymphocytes infiltrating Sjögren’s syndrome (SS) minor salivary glands (MSG) [11].

SS is a chronic systemic autoimmune disease mainly affecting exocrine glands and characterized by autoantibody production and systemic manifestations. Whilst, B cell hyperactivity is central to SS pathogenesis [12], a concomitant role of T cells in disease development and progression has been recognized [13]. The formation of lymphocyte aggregates in MSG is a histological hallmark of SS, and their organization in T and B cell areas reflects lesion severity [14]. Within those aggregates, the development of ectopic germinal centers (GC), characterized by full segregation in T and B cell zones, formation of CD21+ follicular dendritic cell networks has also been described, in association with local B cell proliferation and affinity maturation [15]. This phenomenon is associated with disease severity, autoantibody production, and lymphoma development [16].

Evidence on the role of autophagy in SS is limited. Previous studies pointed at a dysregulation of autophagy in salivary glands of SS mice models [17, 18], and a protective role of this pathway specifically in salivary glands’ epithelial cells following induction of endoplasmic reticulum stress [19]. Nonetheless, the role of autophagy in supporting lymphocyte survival, proliferation, and GC formation in SS is still unknown. Critical unanswered questions pertain to the role of this pathway and its association with the severity of salivary gland infiltrates. This study aims to further explore the role of autophagy in infiltrating and circulating lymphocytes of patients with SS and to investigate its role in disease histopathological progression.

## Materials and methods

### Patients and samples

Unselected consecutive patients with SS (AECG criteria [20]) referring to the Sjögren’s Clinic at Sapienza University of Rome (Italy) were enrolled. Age- and sex-matched healthy subjects were used as control (HC). All of the included subjects gave their written informed consent to participate in the study. Patients’ clinical and laboratory data were recorded, and MSG biopsies were performed for diagnostic purposes. One MSG per patient was collected and snap-frozen at time of biopsy to perform both microdissection analysis and immunofluorescence. Human tonsils belonging to HC treated by tonsillectomy for recurrent tonsillitis were collected and snap-frozen as comparison. From patients with SS and HC, blood samples were collected for peripheral blood mononuclear lymphocyte (PBMC) purification.

### Histological analysis: H&E, immunohistochemistry, and immunofluorescence

Frozen SS MSG were sequentially cut by cryostat (7 μm thick) and evaluated by hematoxylin and eosin (H&E), immunohistochemistry (IHC), and immunofluorescence (IF).

H&E and IHC were used to detect salivary gland infiltrates, to count the number of lymphocytes composing foci, to calculate the focus score, to evaluate aggregates’ composition in T (anti-CD3 Rabbit Polyclonal, DAKO) and B (anti-CD20 Mouse Monoclonal, DAKO) cells, and to identify GC-like structures. In MSG, GC-like structures were defined as organized nodular aggregates resembling the classical secondary lymphoid organs’ GC (with a “light” and “dark” zone) detectable by H&E. GC-like structures were confirmed by staining for CD21+ (CD21L, long isoform) follicular dendritic cells (anti-CD21 Rabbit Polyclonal, DAKO). IHC methods are available in the supplementary materials and previous publications [14]. MSG infiltrates were divided into groups as follows: (1) “small” if composed of 50–100 cells, (2) “large” if composed of more than 100 cells with initial segregation in B and T cell areas but neither resembling a GC-like structure in H&E nor having CD21+ staining, and (3) “GC-like” structures according to the abovementioned definition.

IF analysis was performed on frozen MSG sections and frozen human tonsils sequential to those used for microdissection to characterize the autophagy marker LC3B [NB100-2220 (Novus Biologicals), specific to the autophagosome protein LC3B and recognizing both LC3-I and LC3-II]. CD3+ and CD20+ cells with LC3B expression were identified by visualizing fluorescently labeled “LC3 puncta,” as previously described in other studies [7, 8]. For IF methods, see the supplementary materials. Images were acquired on Zeiss LSM 780 laser-scanning confocal head with a Zeiss Axio Imager Z1 microscope.

### Microdissection and qPCR

Seven-micrometer-thick frozen MSG, sequential to IHC, were cut on Palm Slides in RNase-free condition and stained by Cresyl Violet, in order to perform microdissection by Laser Capture Microdissection Microscope (Zeiss). Using this technique, small infiltrates, large infiltrates, and GC-like structures from MSG and GC from human tonsils were microdissected. On these samples, the expression of autophagy genes Atg5 [ATG5 (Human) Thermo Fisher, Assay ID Hs00169468_m1] and MAP1LC3A [MAP1LC3A (Human) Thermo Fisher, Assay ID Hs01076567_g1] was evaluated by real-time PCR (supplementary materials).

### PBMC isolation and analysis of autophagy in circulating immune cells from HC and SS patients

PBMCs were isolated from HC and SS patients and collected for flow cytometry experiments. Autophagy levels in B and T lymphocytes were detected using Cyto-ID autophagy detection kit (Enzo Life Sciences). The probe used in this kit consists of cationic amphiphilic tracer dye that stains autophagosomes and autophagolysosomes [21]. For the immunophenotyping analysis, see the supplementary materials.

### Statistical analysis

For the statistical analysis, one-way ANOVA, Mann-Whitney *U* test, and Spearman’s rank correlation test were used. When using one-way ANOVA, corrections for multiple comparisons were applied (Dunn’s multiple comparisons test). Two-tailed *p* values < 0.05 were considered significant. Statistical analysis was performed by GraphPad software (version 5.0).

## Results

### Clinical, serological, and histological features of SS patients used for microdissection analysis

Clinical and laboratory features of patients enrolled for microdissection are shown in Table 1 of the supplementary material. In total, 13 MSG and 3 human tonsils were microdissected. The main histological features of MSG were a mean of focus score of 3.9 (SD ± 2.8) and the presence of GC-like structures in 8/20 patients (40%).

### Microdissection of MSG unveils increased expression of autophagy genes in large and more organized infiltrates compared to small

**Fig. 1:**
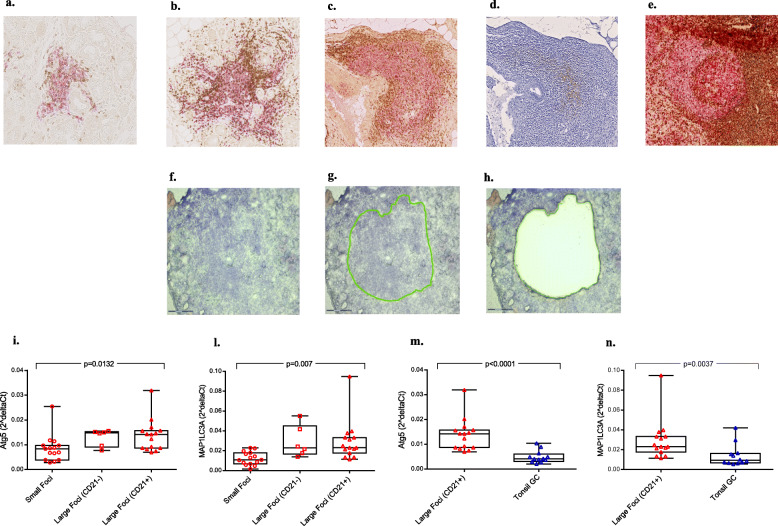
Histological analysis and expression of autophagy markers in SS salivary gland infiltrates. MSG staining for T (CD3+; *brown*) and B (CD20+; *pink*) cells showing two types of foci: “small focus” (**a**) and “large focus (CD21−)” (**b**). MSG IHC staining for T (CD3+; brown) and B (CD20+; pink) lymphocytes (**c**). MSG IHC staining for follicular dendritic cells (CD21; brown) showing a “large focus (CD21+)” (**d**). Tonsil IHC staining for T (CD3+; *brown*) and B (CD20+; *pink*) cells showing a “tonsil GC” (**e**). MSG cresyl violet staining showing a GC-like structure before microdissection (**f**), circumscribed by green line for microdissection (**g**) and following microdissection (**h**). Atg5 and MAPLC3A gene expression levels (2^deltaCt normalized to GAPDH) in three different types of SS salivary gland infiltrates (**i**, **l**). Atg5 and MAPLC3A gene expression levels in large foci CD21+ from SS MSG (GC-like structures) and tonsil GC (**m**, **n**). Data are presented as box and whisker plots; individual data points are shown. *p* values are displayed in each graph; two-tailed unpaired Mann-Whitney *U* test

By microdissection, we collected the following samples: 15 different MSG small foci (Fig. 1a), 6 different MSG large foci CD21− (Fig. 1b), 15 different MSG large infiltrates CD21+ (“GC-like structures”) (Fig. 1c, d), and 12 different GC from tonsils (Fig. 1e). Small infiltrates were obtained from 10 different patients (in 4 cases, more than one small infiltrate was dissected); large CD21− infiltrates were obtained by 5 different patients (in 1 case, more than one was obtained); large CD21+ infiltrates were obtained by 10 different patients (in 3 cases, more than one CD21+ infiltrate was collected). An example of microdissection is shown in Fig. 1f–h.

We found a different expression of autophagy-associated markers between small and large MSG infiltrates. Multiple comparison analysis demonstrated a significant difference in the expression of both Atg5 (*p* < 0.0001) and MAP1LC3A (*p* = 0.0037) between groups, composed by small foci, large foci CD21−, and large foci CD21+ (Fig. 1i, l). Specifically, a significant difference in Atg5 expression between small foci versus large foci (CD21+) and a significant difference in MAP1LC3A expression between small versus large foci (CD21−) and between small versus large foci (CD21+) were identified. No difference in both Atg5 expression and MAP1LC3A expression was identified between large foci CD21+ and large foci CD21−.

A detailed representation of Atg5 and MAP1LC3A expression levels in the different patients is reported in Figure 1a and b of supplementary materials. In two patients, all types of infiltrates (small, large CD21−, and large CD21+) were found in the same salivary gland. For both patients, a higher expression of Atg5 between small and large CD21− foci was identified; in one case, the expression decreased in the large CD21+ focus. Regarding MAP1LC3A, a higher expression in large CD21+ infiltrates compared to small infiltrates was demonstrated in both (Figure 1c and d supplementary materials).

Expression of Atg5 and MAP1LC3A in large CD21+ foci (GC-like structures) from MSG was also higher compared to GC from tonsils (*p* = 0.0001, *p* = 0.0037) (Fig. 1m, n).

### The autophagosome protein LC3B is detectable only in T and B lymphocytes infiltrating MSGs and not in those inhabiting human tonsils

**Fig. 2:**
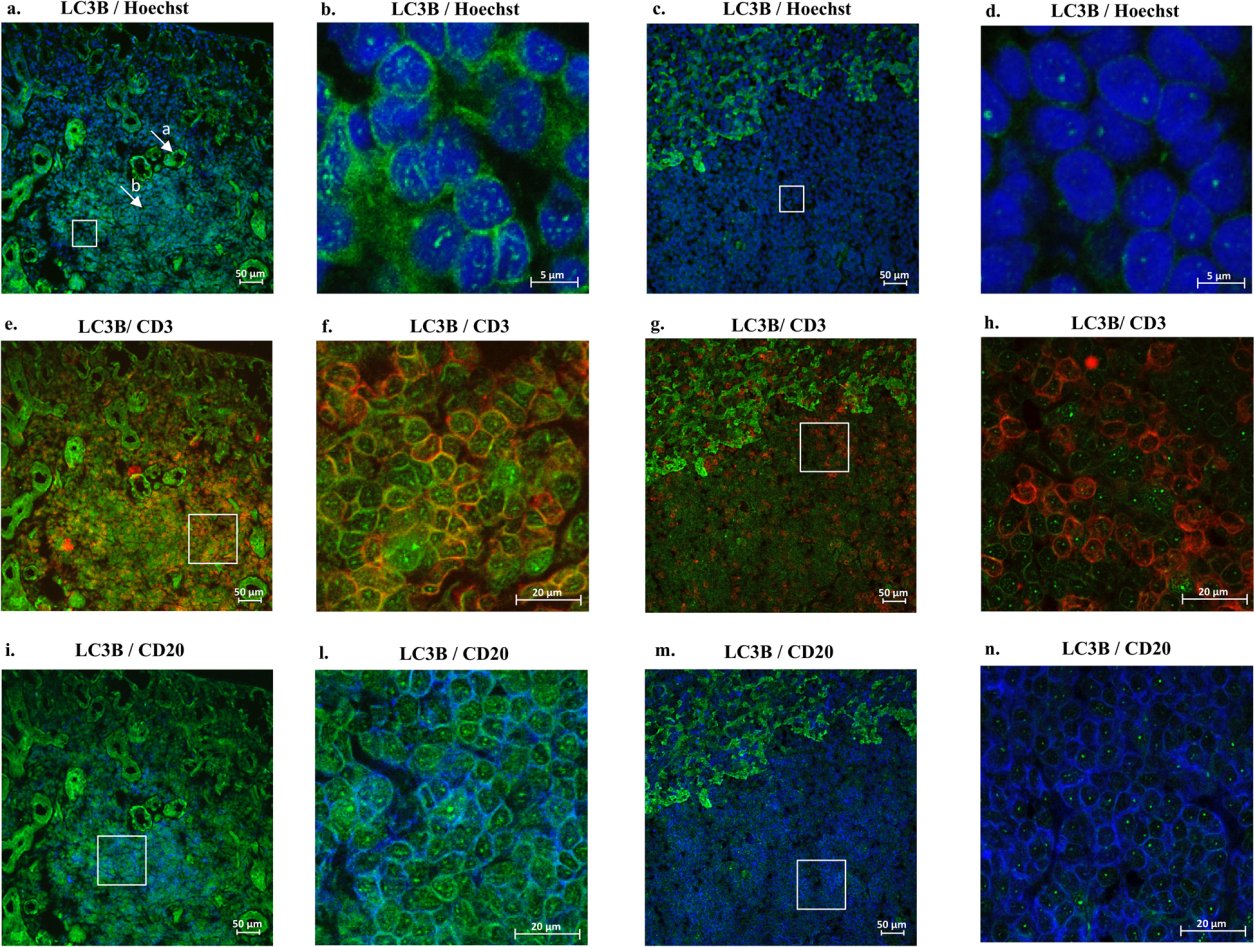
Immunofluorescence in SS minor salivary gland and human tonsil for detection of LC3B-positive lymphocytes. LC3B staining in SS MSG (**a**) [the two white arrows indicate LC3B positive epithelial cells (**a**) and LC3B positive lymphocytes (**b**)] and human tonsil (**c**) [LC3B (*green*); × 25 magnification]. LC3B staining for detection of *LC3 puncta* in cytoplasm of lymphocytes from MSG (**b**) and human tonsil (**d**) [LC3B+ (*green*) and Hoechst stain; × 60 magnification]. Co-localization in MSG [**e** *× 25 magnification* and **f** *× 40 magnification*] and tonsil [**g** *× 25 magnification* and **h** *× 40 magnification*] of autophagy marker LC3B [LC3B+ (*green*)] with T lymphocytes [CD3+ (*red*)]. Co-localization in MSG [**i** *× 25 magnification* and **l** *× 40 magnification*] and tonsil [**m** *× 25 magnification* and **n** *× 40 magnification*] of autophagy marker LC3B [LC3B+ (*green*)] with B lymphocytes [CD20+ (*blue*)]. White squares in **a**, **c**, **e**, **g**, **i**, and **m** indicate the areas that have been magnified [× 60 magnification (**b**, **d**) and × 40 magnification (**f**, **h**, **l**, **n**), respectively]

In MSG, LC3B was diffusely expressed both in ductal epithelial cells and in infiltrates (Fig. 2a). In MSG, lymphocytes expressing the autophagy marker LC3B were visualized by the presence of “LC3 puncta” stain (Fig. 2b—high magnification). In human tonsil, we detected positive staining only at the boundary of the GC (Fig. 2c) and a lower/absent LC3B expression in lymphocytes (Fig. 2d—high magnification), both CD3+ (Fig. 2g, h) and CD20+ (Fig. 2m, n). In MSG, cells showing “LC3 puncta” stain were both CD3+ (Fig. 2e, f) and CD20+ (Fig. 2i, l).

### Immunophenotyping studies revealed no increased expression of autophagy markers in circulating PBMC from patients with SS

Clinical and laboratory features of patients enrolled for the immunophenotyping study are shown in Table 2 of the supplementary material. In total, 30 consecutive SS patients and 20 sex- and age-matched HC were evaluated. Both in patients with SS and HC, immunophenotyping studies revealed higher levels of autophagy in CD3+ cells, compared to CD19+ cells (*p* < 0.0001)] (Figure 2a, b—supplementary material). However, the autophagy level in CD3+ cells from patients with SS was not higher compared to HC (Figure 2c—supplementary material). In HC, the autophagy level in CD19+ cells was slightly higher, compared to SS patients (Figure 2d—supplementary material). No difference in autophagy was detected when stratifying patients, according to the presence or absence of anti-Ro/SSA and anti-La/SSB antibodies, rheumatoid factor, and GC (Table 3—supplementary material). No correlation was observed between autophagy in circulating lymphocytes and focus score (*p* = 0.829, *r* = 0.04).

## Discussion

The present study suggests that autophagy is a feature of T and B cells infiltrating MSG of patients with SS. In comparison to lymphocytes inhabiting secondary lymphoid organs, lymphocytes infiltrating SS MSG display a clear expression of the autophagy marker LC3B, as confirmed by the “LC3 puncta” stain [8] in both CD3+ and CD20+ cells. The presence of upregulated autophagy genes in lymphocytes invading SS MSG could be differently interpreted. Being autophagy a mechanism of cell survival, it is reasonable to hypothesize that during MSG inflammation, this pathway is used by lymphocytes to prevent death and sustain proliferation. Accordingly, upregulation of autophagy genes possibly reflects the presence of lymphocytes with increased lifespan and/or slower turnover due to the concomitant inflammatory activation. In line with this finding, our group recently demonstrated overexpression of molecules favoring lymphocyte survival and proliferation, such as PI3Kδ, especially in large and organized SS MSG infiltrates [22]. Interestingly, in this study, we demonstrated a higher expression of the autophagy genes Atg5 and MAP1LC3A in large and organized infiltrates compared to small infiltrates, thus suggesting that autophagy might be implicated in the organization of salivary gland infiltrates and, possibly, in their severity. Especially for MAP1LC3A, the different expression of autophagy genes according to the size of infiltrates was confirmed also in individual cases. Although this subanalysis was available only for two patients, this finding likely supports the hypothesis that the different expression of autophagy genes between small and large foci is related more to the type of infiltrate than to a specific patient “autophagic state.” This finding is further supported by the variability in autophagy gene expression levels observed among the same type of infiltrate belonging to a singular patient.

Since autophagy is essential for lymphocyte survival, differentiation, and activation [2], as well as for their secretion of inflammatory proteins [23], it is not unexpected that deregulation of this mechanism might be identified in patients with autoimmune conditions, such as SS. In SS, inflammatory infiltrates may differ among patients and their aspect is typically correlated with patients’ serological and clinical features [16]. Large and organized infiltrates are usually associated with the presence of autoantibodies and higher disease activity [16, 24, 25]. Conversely, small and non-organized infiltrates are more commonly observed in seronegative patients with milder disease activity [16]. The evidence of higher autophagy in large and organized infiltrates strongly suggests the involvement of this pathway in their progression.

Our data not only demonstrate that lymphocytes’ autophagy is associated with SS histological severity but also highlight an aberrant regulation of this pathway in SS GC-like structures. Specifically, we identified an overexpression of autophagy compared to the classic GC inhabiting human tonsils, which, as we know, share structural and molecular similarities including the presence of a CD21+ network. Although the definition of GC in SS is still subject of debate, following identification by H&E, the expression of CD21 is largely accepted by the community as a validation of GC-like formation [26]. To this regard, we evaluated autophagy specifically in aggregates displaying both histological appearance of GC and confirmed to express CD21 and we found a significant overexpression in GC-like structures compared to the normal GC. This finding highlights the presence of functional differences between the two structures, highlighting the role of GC-like structures in SS as a pathogenic hub for autoreactive B cell survival, autoantibody production [15], increased systemic disease activity, and more severe disease progression [24, 27]. Thus, the evidence of increased lymphocyte autophagy in these peculiar structures raises the possibility of a role for autophagy in the development of autoimmunity rather than a simple function in supporting lymphoid organization. Nonetheless, the pathogenic role of autophagy in GC formation and maintenance is still unknown. Early studies failed to unveil a role for autophagy in GC of secondary lymphoid organs; however, more recently, a non-canonical autophagy mechanism regulating B cell survival during the GC’ reaction has been described [28]. The presence of GC-like structures in SS MSG is undoubtedly associated with more active and systemic disease [16] and, possibly, with the development of lymphoma [26]. Thus, a relationship between the upregulation of autophagy in SS GC and their pathogenic function is expected.

Of the several effector proteins implicated in autophagy pathways [1], LC3 is commonly utilized as an autophagy marker [5]. In this study, we observed the expression of increased amount of LC3B on both T and B cells infiltrating MSG by the evidence of “LC3 puncta” stain [8]. Interestingly, this finding was not confirmed in lymphocytes inhabiting secondary lymphoid organs.

In T cells, autophagy is involved both in survival homeostatic mechanisms and in their activation [29]. Here, we highlight the presence of autophagy in CD3+ cells infiltrating MSG. In our previous work, we investigated the expression of this pathway specifically on this cell subset identifying a co-localization of Atg5/LC3II markers with CD4+ and CD8+ lymphocytes [11]. However, along SS course, there is also a progressive increase in the foci B cell component [14]. Autophagy is a crucial survival mechanism across differentiation stages of the B cell cycle [30]. In the present study, we demonstrate, for the first time, the presence of autophagic B cells in infiltrating SS MSG.

However, recent studies have suggested that increased expression of cytoplasmic LC3-positive structures may also indicate impaired autophagosome degradation [7], and LC3 puncta stain can be also detected when autophagosome formation is abrogated due to LC3-I sequestration to p62 aggregates which accumulate into the cytoplasm [31]. Thus, the presence of LC3 puncta in lymphocytes infiltrating SS salivary glands also raises the possibility of impaired autophagy. However, taking together data from gene expression analysis and our previous findings [11, 22], we are inclined to consider the high expression of LC3B puncta as result of autophagy activation. Either way, a deregulation of autophagy mechanisms in SS infiltrating lymphocytes is observed and further studies are highly recommended to clarify this aspect.

Although lymphocytes’ autophagy was associated with escalating histological severity, when determining its activation in peripheral circulating lymphocytes, we did not identify any difference compared to HC. The absence of increased autophagy in SS circulating lymphocytes further supports a predominant role of this process at tissue level. Moreover, as pathogenic mechanisms identified with histologic evaluations might be diluted out and not identifiable in the periphery, the lack of differences in peripheral autophagy levels between SS and HC is not unexpected.

## Conclusions

In this study, we prove that autophagy is a feature of lymphocytes infiltrating MSG of patients with SS and we demonstrate a higher expression of autophagy markers in larger and more organized MSG infiltrates. Accordingly, a pathogenic input of autophagy in SS MSG inflammation can be hypothesized. Of note, activation of autophagy is not detectable in circulating lymphocytes from patients with SS reinforcing the idea of a major contribution of this pathway at tissue level rather than in the periphery. Finally, we demonstrate that autophagy is abnormally regulated in SS GC-like structure compared to the classical GC inhabiting secondary lymphoid organs. This finding further increases our knowledge on GC like-structures forming in SS MSG and possibly suggests autophagy as implicated in survival and proliferation of autoreactive lymphocytes.

## Supplementary information

**Additional file 1: Figure 1.** Atg5 and MAP1LC3A gene expression in MSG, relationship between sample infiltrates and patients. **Figure 2.** Flow cytometer analysis of autophagy in circulating T and B lymphocytes shows no difference between patients with SS and HC. **Table 1.** Clinical and serological features of SS patients enrolled for minor salivary glands microdissection. **Table 2.** Clinical, serological and histological features of patients enrolled for PBMC analysis. **Table 3.** Autophagy levels in circulating CD3+ and CD19+ lymphocytes from patients with SS stratified according to the presence of autoantibodies and Germinal Centers.

## Data Availability

All data generated or analyzed during this study are available from the corresponding author on reasonable request.

## Abbreviations

AECG: American-European Consensus Group Criteria
Atg: Autophagy
Atg5: Autophagy-related 5
GCs: Germinal centers
HC: Healthy controls
H&E: Hematoxylin and eosin
IHC: Immunohistochemistry
IF: Immunofluorescence
LC3: Microtubule-associated protein 1A/1B-light chain 3
MAP1LC3A: Microtubule-associated protein 1 light chain 3 alpha
MSG: Minor salivary gland
PBMC: Peripheral blood mononuclear cell
SS: Sjögren’s syndrome
TLS: Tertiary lymphoid structures
