# Supplementary material for: Autophagy occurs in lymphocytes infiltrating Sjögren’s syndrome minor salivary glands and correlates with histological severity of salivary gland lesions
Source: Arthritis Res Ther. 2020 Oct 13;22:238. doi: 10.1186/s13075-020-02317-6 (PMC7557086; doi:10.1186/s13075-020-02317-6)
Supplement: Supplementary file 1 — Additional file 1: Figure 1. Atg5 and MAP1LC3A gene expression in MSG, relationship between sample infiltrates and patients. Figure 2. Flow cytometer analysis of autophagy in circulating T and B lymphocytes shows no difference between patients with SS and HC. Table 1. Clinical and serological features of SS patients enrolled for minor salivary glands microdissection. Table 2. Clinical, serological and histological features of patients enrolled for PBMC analysis. Table 3. Autophagy levels in circulating CD3+ and CD19+ lymphocytes from patients with SS stratified according to the presence of autoantibodies and Germinal Centers. [file 13075_2020_2317_MOESM1_ESM.zip › Supplementary_Material. Revised.docx]

**Supplementary Material.**

**Autophagy occurs in lymphocytes infiltrating Sjögren’s Syndrome minor salivary glands and correlates with histological severity of salivary gland lesions**

Colafrancesco S et al.

**Immunostaining (H&E and Immunohistochemistry) on frozen MSG**

Frozen MSG were cut by cryostat and stained in H&E in order to identify the presence of a Focal Lymphocytic Sialoadenitis (FLS), to identify the presence of foci (focus defined as a lymphocytic infiltrate composed by at least 50 cells), and to calculate the focus score (FS). Immunohistochemistry (IHC) staining were performed with the purpose of detecting infiltrating T and B Lymphocytes as well as FDCs. The following antibodies were used: rabbit polyclonal antibody anti CD3 (DAKO); mouse monoclonal antibody anti-CD20 (DAKO), mouse monoclonal antibody anti-CD21 (DAKO)]. Slides were fixed in 10% PFA and, following the antigen retrieval procedure (high temperature in citrate buffer), peroxidase and protein blocking was performed. For detection of CD3 and CD20 co-localization, the En Vision G/2 Doublestain System DAKO Kit was used. After 1 hour incubation with the primary antibody (anti-CD3), Dextran polymer conjugated with horseradish peroxidase and affinity isolated Immunoglobulins (Polymer/HRP) was incubated for 10 min. DAB + Cromogen was added for CD3+ cells detection. After 5 minutes of Doublestain Block the primary antibody (anti-CD20) was incubated for 1h. After washing, ten minutes of incubation with Dextran polymer coupled with the secondary antibody [Rabbit/Mouse (LINK] and ten minutes of incubation with Dextran polymer conjugated with alkaline phospatase (Polymer/AP) were performed. Permanent Red Chromogen was used for CD20+ cells detection. For detection of CD21+ cells detection, single staining sequential to CD3/CD20 section was performed. After 1 hour incubation with the primary antibody (anti-CD21), Dextran polymer conjugated with horseradish peroxidase and affinity isolated Immunoglobulins (Polymer/HRP) was incubated for 10 min. DAB + Cromogen was added for CD21+ cells detection.

**MSG Image analysis**

Stained slides were scanned by Zeiss Axio Scan. The perimeter of the glandular tissue and foci were manually drawn using Zeiss software.

Focus Score was calculated as follows: [total number of foci/total sg area (mm2)] * 4 mm2;

The following structures were identified:

- Small infiltrates: lymphocyte infiltrates composed by 50-100 cells identified by H&E and then confirmed by CD3+ and CD20+ IHC staining;

- Large infiltrates: lymphocytes infiltrates composed by more than 100 cells identified by H&E and then confirmed by CD3+ and CD20+ IHC staining with evidence of an initial segregation in B and T cells areas, but negative for CD21+ staining;

- GCs-like structures: lymphocyte infiltrate composed by more than 100 cells identified by H&E with evidence of a “dark zone” and “light zone”; confirmed by CD3+ and CD20+ IHC staining with evidence of segregation in B and T cells areas and CD21+ staining.

**Immunofluorescence staining on MSG**

MSG frozen sections of 6µm in thickness were cut, left to dry overnight at room temperature and next day stored at -80° until use. For IF analysis, slides were allowed to reach room temperature and then fixed for 20 minutes in ice-cold acetone, left to dry and then hydrated in PBS 1x. For the staining, all dilutions of reagents and antibodies were made in PBS 1x with 1% BSA (Sigma). Sections were first treated with PBS 1x-Triton 0.05% for 5 minutes, followed by blocking with 10% horse serum. Slides were incubated for 120 minutes with ‘cocktails’ containing the following primary antibodies: anti-CD3 and anti-CD20 (DAKO) and anti-human-LC3B (NB100-2220, Novus Biologicals). Hoechst (Molecular Probes) was used for nuclear stain. All secondary antibodies (IgG alexa fluor 488 for LC3II/Atg5; IgG1 TRITC for CD3; IgG2 Cy5 for CD20) were incubated for 30 minutes. Slides were mounted with Prolong Gold Antifade reagent (Invitrogen Life Technologies). Images were acquired on Zeiss LSM 780 laser-scanning confocal head with a Zeiss Axio Imager Z1 microscope; LSM510 Image Examiner Software was used to process the images.

**MSG sectioning and Microdissection procedure**

In RNA free condition, 7-micron thick frozen MSG sections, sequential to those stained in H&E and IHC, were cut on Palm Slides. Palm slides (Membrane slides 1.0 PEN NF 41590-9081-000) were previously sterilised and activated by UV light. Salivary gland sections were taken from the cutting level at which cellular infiltration was detected by Toluidine blue staining. Sections were sequentially cut both on normal slides (for H&E and IHC staining) and on Palm Slides. Once finished, Palm Slides were placed into a box with dry ice and then stored in a slide boxes at −80 °C until required. With the same procedure, sections of frozen human tonsil were cut and used as control for microdissection.

A falcon tubes (50ml) with cresyl violet acetate (SIGMA C5042) at 0.1% w/v in 100% ACS grade ethanol were prepared and incubated overnight at room temperature under constant agitation. Cresyl violet staining were performed both on salivary glands and on human tonsils (control). After removing the slides from the -80°, they were sequentially dived into: 70% Ethanol for 2 minutes, Cresyl violet solution for 20 seconds, 70% Ethanol for 2 minutes and 100% Ethanol two times 2 minutes each. After drying, slides were transferred to a new box filled with dry ice and stored back at -80° until dissection.

Laser Microdissection (Leica Microsystem), also known as LMD or LCM (Laser Capture Microdissection), is a contact- and contamination-free method for isolating specific single cells or entire areas of tissue from a wide variety of tissue samples. Frozen slides were removed from the box with dry ice. Once completely dry, Palm Slides were put one by on the slide-stack holder and microdissected by laser in order to pick up and collect the following infiltrates: small MSG infiltrates, large MSG infiltrates (CD21 negative in IHC), GCs from MSG (CD21 positive in IHC), GCs from human tonsils.

For each of the above-mentioned structures at least 6 sequential slides of the same infiltrate/GC were collected in micro tubes pre filled with lysis buffer; after collection microtubes were stored back at -80° until qPCR.

**Quantitative RT-PCR on microdissected samples**

RNA was extracted from microdissected samples using the Qiagen RNeasy Micro kit, according to manufacturer’s instructions. Tissues stored at -80°C were defrosted on ice, and 200ul of RLT buffer (containing 10ul of β-Mercaptethanol/ ml of buffer) were added to each tube. The tissue lysis was carried out by vortexing each sample tube for at least 30-50 seconds until the sample was uniformly homogeneous. Tissue lysates were centrifuged very briefly at maximum speed (13000rpm) to collect the sample at the bottom of the tube. An equal volume of 70% ethanol was added to the lysate and immediately mixed by pipetting in order to precipitate RNA, which remains in the aqueous phase. The sample (400ul) were added to the RNeasy mini column placed in a 2 ml collection tube and centrifuged for 1 minute at 13,000 rpm to allow the RNA to bind to the silica column. The flow-through was discarded, and 350 μl of RW1 Buffer were added to the column, centrifuged for 1 minute at 13,000 rpm and the flow-through discarded. To avoid any possible DNA contamination, a DNase step was included according to manufacturer’ instructions.10 μl of DNAse I stock solution (previously prepared by dissolving solid DNase I (1500 Kunitz units) in 550 μl of RNase-free water) were added to 70μl of Buffer RDD, gently mixed and added to the RNeasy mini column silica-gel membrane. Following 15 min incubation at room temperature (RT), 350 μl of Buffer RW1 were added to the column, centrifuged for 1 minute at 13,000 rpm and the flow-through discarded. The RNeasy column was transferred into a new 2 ml RNase free collection tube. In order to wash away contaminants (residual DNA and proteins) in the organic phase, 500 μl of Buffer RPE were placed onto the RNeasy column and centrifuged for 1 minute at 13,000 rpm to wash the column. The flow-through was then discarded and another 500 μl Buffer RPE added to the RNeasy column. The tube was centrifuged for 2 min at 13,000 rpm to dry the RNeasy silica-gel membrane and the flow-through discarded. To eliminate any chance of possible Buffer RPE carryover, the tube was centrifuged again for 1 min at 13,000 rpm. For elution, the RNeasy column was transferred to a new RNAse free 1.5 ml collection tube, and 25 μl of RNase-free water were pipetted directly onto the RNeasy silica-gel membrane and the tube centrifuged for 1 min at 13,000 rpm. The eluted RNA for each sample was immediately frozen at -80°C until required.

Reverse transcription was carried out using the High capacity reverse transcription cDNA synthesis kit (Applied biosystems) according to the manufacturer’s guidelines in a 96 well plate. Briefly, 25ul of total RNA for each sample was mixed with 5 ul 10x Buffer, 5ul 10x Random Primers, 2ul 100nM dNTPs, 2.5ul Reverse transcriptase enzyme and brought to 50ul volume reaction with DNase/RNase-free water. After mixing and a brief spin down of the 96 well plate, the reverse transcription to cDNA was carried out on Techne 312 Thermal Cycler PCR machine using the following conditions: 25ºC for 10 min and 37ºC for 120 min. Finally, the completed cDNA was diluted with 50ul DNase/RNase-free water in a 1:1 ratio. The cDNA was then stored at -20ºC until further use.

Relative expression of Atg5 and MAP1LC3 genes was quantified by TaqMan RT-PCR. Reactions were carried out in a 384-well optical reaction PCR plate (Applied Biosystems) in a final volume of 5.1μl. The reaction mix contained a forward primer, a reverse primer and a probe, specific to the gene of interest or the housekeeping gene (GADPH). Already published TaqMan probes and primers from Applied Biosystems were used. These were diluted to their optimal pre-determined working concentrations. Probes for both the house-keeping and target gene were labelled with FAM. 2.8μl of Taqman gene expression master mix (Applied Biosystems) was also added to each reaction, along with 2.3μl of cDNA template. Only for microdissected samples, before running the reaction, the plate was sealed using a MicroAmp™ Clear Adhesive Film (Applied Biosystems) and centrifuged for 1 min at 350 x g. The quantitative real-time PCR were run in duplicates per gene for each sample and detected by the ABI PRISM 7900HT Instrument. The standard thermal cycling conditions were used that comprised of a 2 min UNG activation step at 50 ºC, at 95ºC. Taq polymerase enzyme activation step for 10 min, and cycles of 95 ºC denaturation for 15 sec and 60 ºC anneal/extension for 60 sec. Results were then analysed after 40 cycles of amplification using the ABI PRISM 7900HT Sequence Detection System Version 2.1 (SDS 2.1). Cycle thresholds (Ct) were determined within the logarithmic phase of the PCR for the house-keeping gene and the target gene. GADPH was used as an endogenous control. We used the mean of two technical replicates (Ct values) to calculate the ΔCt value for which the Ct of the GADPH was subtracted from the Ct of the target gene. Ct value and the relative amount was calculated as 2−ΔCt. Ct values above 34 were not accepted, and neither were technical replicates with more than two cycle differences between them.

**Immunophenotyping analysis of circulating lymphocytes**

PBMCs (1×106 cells for each condition) were washed in PBS and stained with allophycocyanin (APC)-conjugated anti-CD3 and fluorescein isothiocyanate (FITC)-conjugated anti-CD19 antibodies (Miltenyi Biotec). After 20 minutes on ice, cells were incubated with CYTO-ID® Green Detection Reagent for 30 minutes in the dark, at 37 °C. 100,000 events per sample were run on FACSCalibur flow cytometer (BD Biosciences) and data were analyzed using the Cell Quest Pro software (BD Biosciences). Values were shown as mean fluorescence intensities (MFI) of Cyto-ID.

**Figure Legend supplementary material.**

**Fig. 1**

**Atg5 and MAP1LC3A gene expression in MSG, relationship between sample infiltrates and patients.**

Atg5 (a) and MAP1LC3A (b) gene expression levels (2^deltaCt normalised to GAPDH) according to patients and type of infiltrates. Each sample is represented as a circle (small infiltrates), square (large CD21) or triangle (large CD21+) and each colour refers to one specific patient. Evaluation of Atg5 (c) and MAP1LC3A (d) gene expression levels (2^deltaCt normalised to GAPDH) in three different types of infiltrates belonging to 2 patients [Patient 1 (Pt 1) and Patient 2 (Pt2)]. For each patient, when more than one sample for a specific type of infiltrate was present, the reported value (represented as a circle, square or triangle) refers to the mean expression level of Atg5 (c) and MAP1LC3 (d).

**Fig. 2**

**Flow cytometer analysis of autophagy in circulating T and B lymphocytes shows no difference between patients with SS and HC.**

Autophagy levels in circulating CD3+ and CD19+ lymphocytes from patients with SS (a) and HC (b). Autophagy levels in CD3+ cells (c) and CD19+ cells (d) in patients with SS compared to HC. Data are presented as box and whiskers plots and values, shown as mean fluorescence intensities (MFI) of Cyto-ID, are shown as points (red for SS and blue for HC). P-values are displayed in each graph; two-tailed unpaired Mann-Whitney U test.

**TABLE 1 (Supplementary Material)**

**Clinical and serological features of SS patients enrolled for minor salivary glands microdissection.**

|  | **SS features**  **(n=20)** | **SS without**  **Germinal Centers (n=8)** | **SS with**  **Germinal Centers (n=12)** |
| --- | --- | --- | --- |
| Age, y (mean ± SD) | 52.5 ± 16.3 | 56.1 ± 16.9 | 48.5 ± 16.0 |
| Female/Male (n°/n°) | 18/2 | 6/2 | 12/0 |
| ESSDAI (mean ± SD) | 1.2 ± 1.8 | 0.57 ± 0.97 | 1.81 ± 2.22 |
| ANA, n°/tot (%) | 13/20 (65) | 4/8 (50) | 8/12 (66.6) |
| Anti-Ro/SSA, n°/tot (%)*** | 11/20 (55) | 3/8 (37.5) | 7/12 (58.3) |
| Anti-La/SSB, n°/tot (%) | 8/20 (40) | 1/8 (12.5) | 6/12 (50) |
| Rheumatoid Factor, n°/tot (%) | 4/20 (20) | 1/8 (12.5) | 4/12 (33.3) |
| Hypergammaglobulinaemia*, n°/tot (%) | 7/20 (35) | 2/8 (25) | 5/12 (41.6) |
| Cryoglobulinemia, n°/tot (%) | 0/20 (0) | 0/0 (0) | 0/0 (0) |
| Monoclonal Component, n°/tot (%) | 1/20 (5) | 1/8 (12.5) | 0/12 (0) |
| Hypocomplementaemia **, n°/tot (%) | 2/20 (10) | 0/8 (0) | 2/12 (16.6) |
| Focus score (mean ± SD) | 3.9 ±2.8 | 2.26 ± 1.24 | 4.98 ± 3.25 |
| Germinal Centers, n°/tot (%) | 12/20 (60) | 0/8 (0) | 12/12 (100) |

*LEGEND:*

*ESSDAI: Eular Sjögren Syndrome Disease Activity Score; ANA: anti-nuclear antibodies; *Hypergammaglobulinaemia: immunoglobulins >16 g/l; **Hypocomplementaemia: C3<80 mg/dl; C4<15 mg/dl; ***anti-Ro/SSA include both anti-Ro52 and anti-Ro60.*

**TABLE 2 (Supplementary Material)**

**Clinical, serological and histological features of patients enrolled for PBMC analysis.**

|  | **SS features**  **(n=30)** | **HC features**  **(n=20)** |
| --- | --- | --- |
| Age, y (mean ± SD) | 59.2 ± 13.4 | 44.6 ± 10.04 |
| Female/Male (n°/n°) | 29/1 | 18/2 |
| ESSDAI (mean ± SD) | 0.6 ± 1.2 | - |
| ANA, n°/tot (%) | 20/30 (66.6) | 0/20 (0) |
| Anti-Ro/SSA, n°/tot (%)*** | 14/30 (46.6) | 0/20 (0) |
| Anti-La/SSB, n°/tot (%) | 8/30 (26.6) | 0/20 (0) |
| Rheumatoid Factor, n°/tot (%) | 5/30 (16.6) | 0/20 (0) |
| Hypergammaglobulinemia, n°/tot (%)* | 6/30 (20) | 0/20 (0) |
| Cryoglobulinemia, n°/tot (%) | 0/30 (0) | 0/20 (0) |
| Monoclonal Component, n°/tot (%) | 2/30 (6.6) | 0/20 (0) |
| Hypocomplementemia, n°/tot (%)** | 0/30 (0) | 0/20 (0) |
| Focus Score, mean ± SD | 3.5 ± 1.86 | - |
| Germinal Centers, n°/tot (%) | 15/30 (50) | - |

*LEGEND:*

*ESSDAI: Eular Sjögren Syndrome Disease Activity Score; ANA: anti-nuclear antibodies; *Hypergammaglobulinaemia: immunoglobulins >16 g/l; **Hypocomplementaemia: C3<80 mg/dl; C4<15 mg/dl; ***anti-Ro/SSA include both anti-Ro52 and anti-Ro60.*

**TABLE 3**

**Autophagy levels in circulating CD3+ and CD19+ lymphocytes from patients with SS stratified according to the presence of autoantibodies and Germinal Centers.**

|  | **CD3+** | | **CD19+** | |
| --- | --- | --- | --- | --- |
| Anti Ro/SSA + (mean ± SD)  Anti Ro/SSA - (mean ± SD) | 15.7 ± 5.04  18.5 ± 3.6 | p=ns | 6.9 ± 2.5  7.9 ± 3.2 | p=ns |
| Anti-La/SSB + (mean ± SD)  Anti La/SSB - (mean ± SD) | 14.5 ± 3.0  17.1 ± 6.2 | p=ns | 6.6 ± 2.6  7.7 ± 3.08 | p=ns |
| Rheumatoid Factor + (mean ± SD)  Rheumatoid Factor - (mean ± SD) | 15.7 ± 3.4  15.2 ± 5.4 | p=ns | 8.2 ± 4.4  7.0 ± 2.6 | p=ns |
| Germinal Center + (mean ± SD)  Germinal Center – (mean ± SD) | 17.3 ± 6.2  15.5 ± 5.0 | p=ns | 7.4 ± 3.4  7.2 ± 2.4 | p=ns |
