# Supplementary figures and images for: Autophagy occurs in lymphocytes infiltrating Sjögren’s syndrome minor salivary glands and correlates with histological severity of salivary gland lesions
Source: Arthritis Res Ther. 2020 Oct 13;22:238. doi: 10.1186/s13075-020-02317-6 (PMC7557086; doi:10.1186/s13075-020-02317-6)

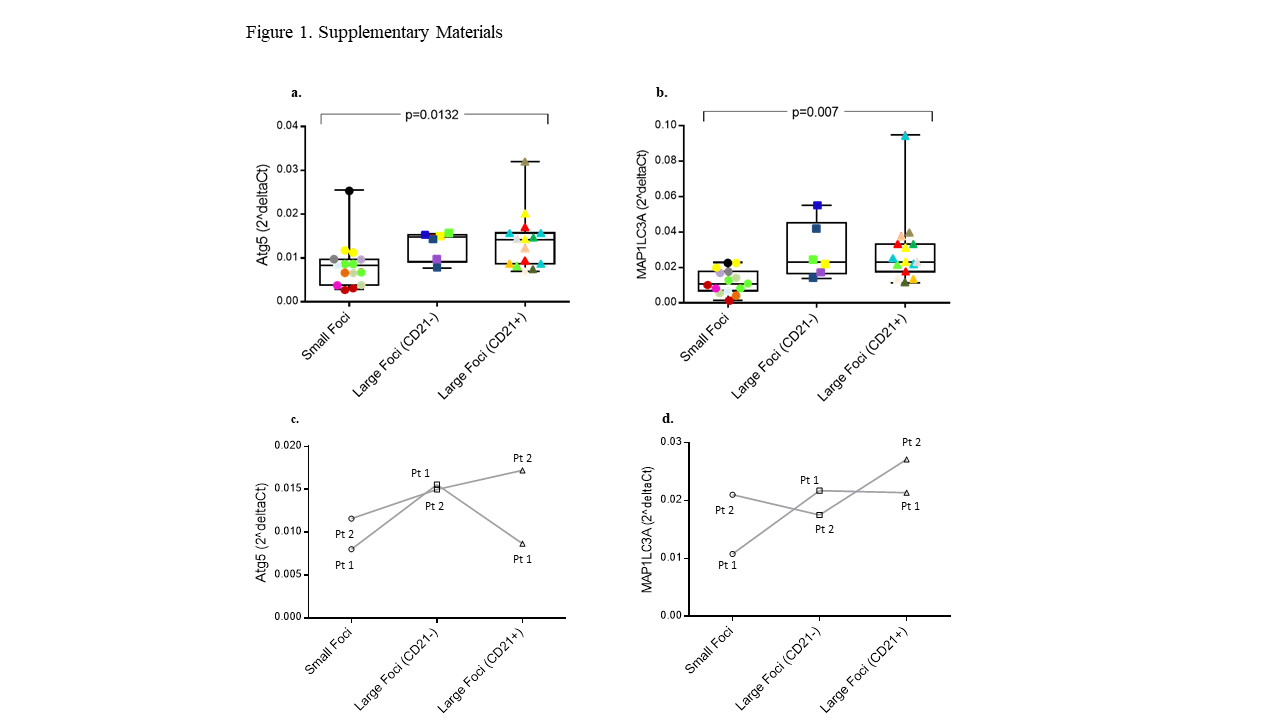

Supplement: Supplementary file 1 — Additional file 1: Figure 1. Atg5 and MAP1LC3A gene expression in MSG, relationship between sample infiltrates and patients. Figure 2. Flow cytometer analysis of autophagy in circulating T and B lymphocytes shows no difference between patients with SS and HC. Table 1. Clinical and serological features of SS patients enrolled for minor salivary glands microdissection. Table 2. Clinical, serological and histological features of patients enrolled for PBMC analysis. Table 3. Autophagy levels in circulating CD3+ and CD19+ lymphocytes from patients with SS stratified according to the presence of autoantibodies and Germinal Centers. [file 13075_2020_2317_MOESM1_ESM.zip › Figure 1. Supplementary Material.tif]

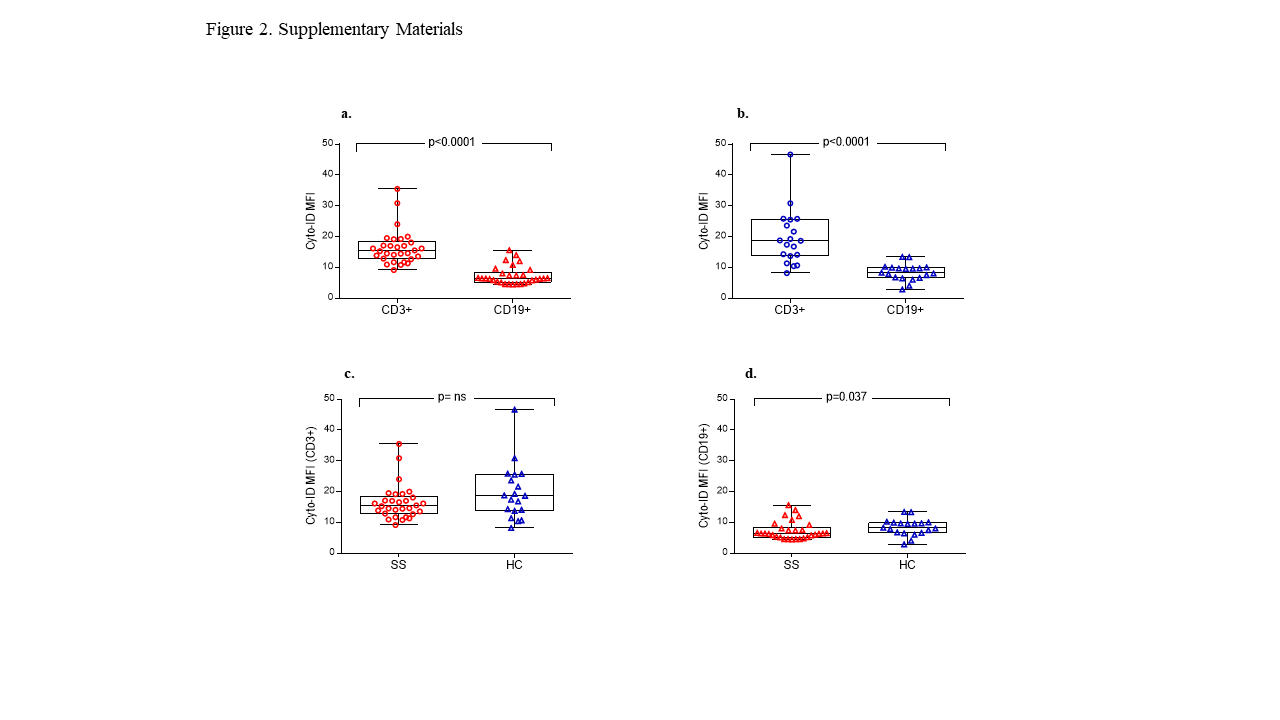

Supplement: Supplementary file 1 — Additional file 1: Figure 1. Atg5 and MAP1LC3A gene expression in MSG, relationship between sample infiltrates and patients. Figure 2. Flow cytometer analysis of autophagy in circulating T and B lymphocytes shows no difference between patients with SS and HC. Table 1. Clinical and serological features of SS patients enrolled for minor salivary glands microdissection. Table 2. Clinical, serological and histological features of patients enrolled for PBMC analysis. Table 3. Autophagy levels in circulating CD3+ and CD19+ lymphocytes from patients with SS stratified according to the presence of autoantibodies and Germinal Centers. [file 13075_2020_2317_MOESM1_ESM.zip › Figure 2. Supplementary Material.tif]
